# *Lactococcus lactis* ZB2 from Zhejiang fermented bamboo shoots enhance intestinal barrier and immune homeostasis in mice

**DOI:** 10.3389/fmicb.2026.1847505

**Published:** 2026-06-24

**Authors:** Ting Xu, Rihui Lu, Yuchen Shi, Yongjin Fang, Hongqiang Lou

**Affiliations:** 1 Department of Nosocomial Infection Management, The Affiliated Hospital of Jinhua University of Vocational Technology, Jinhua, China; 2 Department of Otolaryngology Head and Neck Surgery, Jinhua Municipal Central Hospital, Jinhua, China; 3 School of Medicine, Jinhua University of Vocational Technology, Jinhua, China

**Keywords:** fermented bamboo shoots, gut microbiota, immune homeostasis, intestinal barrier function, *Lactococcus lactis*, short-chain fatty acids

## Abstract

Traditional fermented foods harbor functionally diverse microbial communities that represent an underexplored source of probiotic strains. Lactic acid bacteria were systematically isolated from traditionally fermented bamboo shoots collected across Zhejiang Province, China, with the aim of identifying superior probiotic candidates. Among the isolates recovered, *Lactococcus lactis* ZB2 demonstrated robust gastrointestinal tolerance, favorable adhesion properties, and broad-spectrum antimicrobial activity, consistently matching or exceeding the reference strain *Lactobacillus rhamnosus* GG across all evaluated *in vitro* criteria. Molecular identification via 16S rRNA gene sequencing confirmed ZB2 as *L. lactis subsp. lactis* (>99.7% sequence identity). To evaluate its *in vivo* effects, ZB2 (1 × 10^9^ CFU/day, 200 μL of bacterial suspension in 0.01 M PBS, pH 7.4) was administered by oral gavage to healthy C57BL/6 J mice for 28 days. ZB2 supplementation significantly reinforced intestinal epithelial barrier integrity, evidenced by reduced serum permeability markers (FITC-dextran flux, DAO, D-lactic acid, and LPS), upregulation of tight junction proteins (*ZO-1, Occludin, Claudin-1*) and the mucin gene *Muc2*, and a significant increase in goblet cell number per crypt. Immune homeostasis was modulated toward an anti-inflammatory phenotype, characterized by elevated serum IL-10 and TGF-*β*, reduced IFN-*γ*, downregulation of colonic pro-inflammatory cytokines (*Tnf-α, Il-6, Il-1β*), and upregulation of the antimicrobial peptides *Reg3γ* and *β-defensin 1*. 16S rRNA amplicon sequencing revealed selective enrichment of butyrate-producing genera within the Lachnospiraceae family, accompanied by marked increases in fecal short-chain fatty acid concentrations—particularly butyrate and propionate. Systemic antioxidant capacity was also enhanced, as reflected by elevated SOD, CAT, and GSH-Px activities and reduced malondialdehyde. These findings support the characterization of *L. lactis* ZB2 as a multifunctional probiotic candidate and highlight Zhejiang fermented bamboo shoots as a valuable, underexplored reservoir of superior probiotic strains with broader functional food development potential.

## 1 Introduction

Lactic acid bacteria (LAB) from traditional fermented foods have attracted growing interest as sources of novel probiotic strains with demonstrable health benefits. These foods, produced through microbial fermentation processes that have evolved over centuries, harbor diverse lactic acid bacteria communities that not only preserve food matrices but also confer remarkable functional benefits to the host (Xu et al., 2022; Valentino et al., 2024). Among these traditional fermented products, fermented bamboo shoots occupy a distinctive position as a region-specific delicacy widely consumed across Asian countries, particularly in southwestern China and northeastern India (Behera and Balaji, 2021; Long et al., 2023). Rich in LAB populations dominated by genera such as *Lactobacillus*, *Lactococcus*, and *Leuconostoc*, fermented bamboo shoots have demonstrated anti-inflammatory, antioxidant, and immunomodulatory properties (Singhal et al., 2021; Das et al., 2023). However, despite their widespread consumption and recognized health benefits, the probiotic potential of LAB strains isolated from Zhejiang fermented bamboo shoots remains largely unexplored. Strains adapted to the unique fermentation environment may possess distinctive probiotic characteristics, including enhanced tolerance to gastrointestinal stresses and superior colonization capabilities, making them promising candidates for functional food development and therapeutic applications (Kanpiengjai et al., 2022; Pyo et al., 2024).

The intestinal barrier–immune–microbiota axis underpins host health by coordinating interactions that govern overall physiological homeostasis (Liu et al., 2020; Gou et al., 2022). The intestinal epithelial barrier, comprising tight junction proteins (zonula occludens-1, occludin, claudin-1) and mucin secretions, serves as the primary physical defense against luminal pathogens and toxins while simultaneously regulating nutrient absorption and immune surveillance (Liu et al., 2020; Han et al., 2023). Disruption of this barrier integrity has been implicated in numerous pathological conditions, including inflammatory bowel disease, metabolic disorders, and systemic inflammation (Di Vincenzo et al., 2024; Neurath et al., 2025). Probiotics exert multidimensional beneficial effects through several well-established mechanisms: strengthening physical barrier function via upregulation of tight junction protein expression and mucin production (Liu et al., 2021; Mercado-Monroy et al., 2026); modulating immune homeostasis by balancing pro-inflammatory and anti-inflammatory cytokine profiles; reshaping gut microbiota composition through competitive exclusion of pathogens and enrichment of beneficial taxa such as *Akkermansia*, *Bifidobacterium*, and *Faecalibacterium* (Zheng et al., 2023; Di Vincenzo et al., 2024); and enhancing metabolic capacity through increased production of short-chain fatty acids (SCFAs) including acetate, propionate, and butyrate (de Almeida Brasiel and Luquetti, 2025). Among probiotic genera, *Lactococcus lactis* has garnered considerable attention due to its Generally Recognized as Safe (GRAS) status, robust technological properties, and demonstrated efficacy in reinforcing intestinal barrier integrity and immunomodulation (Tenea and Suárez, 2020; De Chiara et al., 2024; Padilla-García et al., 2025). Recent studies further suggest that *L. lactis* strains, their derivatives, or *L. lactis*-containing formulations can modulate inflammatory signaling, including TLR4/NF-κB-related pathways, and may engage the AMPK/MLCK-tight-junction axis, thereby alleviating intestinal inflammation and barrier dysfunction (Liu et al., 2024; Liu et al., 2025). The systematic screening and characterization of superior probiotic strains is therefore essential for advancing functional food development and precision nutrition strategies.

Building upon this foundation, the present study was designed to address a critical knowledge gap in probiotic resource exploitation from traditional fermented foods. We aimed to systematically isolate and characterize lactic acid bacteria from Zhejiang fermented bamboo shoots, evaluate their probiotic potential through comprehensive *in vitro* screening assays (acid/bile tolerance, epithelial adhesion, antimicrobial activity, and safety profiles), and identify superior strains via molecular approaches. Subsequently, selected *Lactococcus lactis* strains were investigated in a healthy murine model to elucidate their health-promoting effects, with particular emphasis on intestinal barrier reinforcement, immune homeostasis modulation, gut microbiota restructuring, and metabolic regulation. This integrated strategy seeks to provide robust scientific evidence for the functional exploitation of traditional fermented bamboo shoots and establish a theoretical framework for developing next-generation probiotic interventions targeting preventive health management.

## 2 Materials and methods

### 2.1 Sample collection and bacterial isolation

Traditionally fermented bamboo shoot samples were collected from five representative households and local markets in Zhejiang Province, China. Approximately 25 g of each sample was homogenized in 225 mL of sterile 0.85% (w/v) NaCl solution and serially diluted (10-fold). Aliquots (100 μL) of appropriate dilutions were plated onto de Man, Rogosa and Sharpe (MRS) agar (Oxoid, UK) supplemented with 0.5% (w/v) CaCO3 and incubated anaerobically at 37 °C for 48–72 h. Colonies displaying distinct morphology (smooth, white, opaque) surrounded by clear halos indicative of acid production were selected. Each isolate was purified by three successive transfers on MRS agar and stored in MRS broth containing 20% (v/v) glycerol at −80 °C until further use.

### 2.2 *In vitro* probiotic characterization

#### 2.2.1 Tolerance test

Acid tolerance was assessed by resuspending mid-log-phase bacterial cells (approximately 10^9^ CFU/mL) in phosphate-buffered saline (PBS) adjusted to pH 2.0 or pH 3.0 with 1 M HCl. Cultures were incubated at 37 °C for 3 h with gentle agitation. Viable cell counts were determined by plating on MRS agar before and after exposure. Survival rate was calculated as follows: Survival rate (%) = (log CFU/mL after treatment / log CFU/mL before treatment) × 100.

Bile salt tolerance was evaluated by inoculating isolates into MRS broth supplemented with 0.3, 0.5%, or 1.0% (w/v) ox gall (Sigma-Aldrich, United States). Following incubation at 37 °C for 3 h under anaerobic conditions, survival rates were determined as described above. Isolates exhibiting survival rates exceeding 70% at pH 2.0 and 60% in 1.0% bile salt were considered tolerant and advanced for further evaluation.

To establish a comprehensive evaluation of gastrointestinal tolerance, the tolerance of selected strains to simulated intestinal fluid (SIF) was additionally assessed. SIF was prepared according to the United States Pharmacopeia protocol by dissolving 6.8 g KH₂PO₄ in 250 mL distilled water, adjusting to pH 6.8 with 0.2 M NaOH, adding pancreatin (Sigma-Aldrich, United States) to a final concentration of 1 mg/mL, and sterilizing by filtration through 0.22-μm membranes. Bacterial cells were resuspended in SIF at approximately 10^9^ CFU/mL and incubated at 37 °C for 0, 1, 2, and 3 h under anaerobic conditions. Viable cell counts were determined at each time point by serial plating on MRS agar.

#### 2.2.2 Colonization capacity assessment: cell surface hydrophobicity, auto-aggregation, and co-aggregation

Since intestinal epithelial cell culture facilities were unavailable, the colonization potential of selected isolates was evaluated using three well-established physicochemical and microbiological proxy assays: cell surface hydrophobicity, auto-aggregation ability, and co-aggregation ability with enteric pathogens. These parameters serve as widely accepted *in vitro* surrogates for mucosal adhesion capacity in probiotic screening studies (Darmastuti et al., 2021; Vergalito et al., 2024).

##### 2.2.2.1 Cell surface hydrophobicity

Cell surface hydrophobicity was determined using the Microbial Adhesion to Hydrocarbons (MATH) method originally described by Rosenberg (2006) with minor modifications. Bacterial cells harvested at mid-log phase were washed twice with PBS (pH 6.2) and resuspended in the same buffer to an OD₆₀₀ of 0.4–0.6 (A₀). A 3 mL aliquot of the bacterial suspension was vortexed vigorously with 1 mL of n-hexadecane (Sigma-Aldrich, United States) for 2 min, followed by equilibration at room temperature for 15 min to allow complete phase separation. The absorbance of the aqueous phase was then measured at 600 nm (A₁). Hydrophobicity was calculated as:


$$\mathrm{Hydrophobicity}\;\left(\%\right)=\left(1-A₁/\mathrm{A0}\right)\times 100$$

##### 2.2.2.2 Auto-aggregation ability

Auto-aggregation assays were performed according to the method of Kos et al. (2003) with minor modifications. Bacterial suspensions were prepared in PBS (pH 7.2) at approximately 10^8^ CFU/mL, and the initial absorbance was recorded at 600 nm (A₀). Suspensions were incubated statically at 37 °C, and the absorbance of the upper phase (1 mL, withdrawn without disturbing the sediment) was measured at 2 h (A₂) and 24 h (A₂₄) intervals. Auto-aggregation was expressed as:


$$\mathrm{Auto}-\mathrm{aggregation}\;\left(\%\right)=\left(1-At/\mathrm{A0}\right)\times 100$$

where A_t_ represents the absorbance at the respective time point. All experiments were performed in biological triplicate on three independent occasions.

##### 2.2.2.3 Co-aggregation ability

Co-aggregation between selected probiotic isolates and the enteric pathogens *Escherichia coli* ATCC 25922, *Staphylococcus aureus* ATCC 25923, and *Salmonella Typhimurium* ATCC 14028 was evaluated according to the method of Collado et al. (2008) with modifications. Bacterial and pathogen suspensions were independently prepared in PBS (pH 7.2) at 10^8^ CFU/mL and adjusted to A₀ = 0.5 ± 0.02 at 600 nm. Equal volumes (1.5 mL each) of the probiotic and pathogen suspensions were mixed, vortexed for 10 s, and incubated statically at 37 °C for 4 h. The absorbance of the upper phase was subsequently measured (A_mix). Monoculture controls of each strain were measured in parallel (A_prob and A_path, respectively). Co-aggregation was calculated as:


$$\begin{array}{l} \mathrm{Co}-\mathrm{aggregation}\;\left(\%\right)=\left[\left(A\_\mathrm{prob}+A\_\mathrm{path}\right)/2-A\_\mathrm{mix}\right]/\\ \left[\left(A\_\mathrm{prob}+A\_\mathrm{path}\right)/2\right]\times 100 \end{array}$$

All assays were conducted in biological triplicate, and *Lactobacillus rhamnosus* GG ATCC 53103 was included as a reference probiotic strain.

#### 2.2.3 Antimicrobial activity

Antimicrobial activity against pathogenic indicator strains—*Escherichia coli* ATCC 25922, *Staphylococcus aureus* ATCC 25923, *Salmonella typhimurium* ATCC 14028, and *Listeria monocytogenes* ATCC 19111—was assessed using the agar well diffusion method. Briefly, indicator strains grown to mid-log phase were spread uniformly on Mueller-Hinton agar plates. Wells (6 mm diameter) were punched aseptically and filled with 100 μL of cell-free culture supernatant (CFCS) obtained by centrifugation at 10,000 × g for 15 min, followed by filtration through 0.22-μm membranes. Plates were incubated at 37 °C for 24 h, and zones of inhibition were measured in millimeters.

### 2.3 Molecular identification and phylogenetic analysis

Genomic DNA of selected strains was extracted using a commercial bacterial DNA extraction kit (TIANamp Bacteria DNA Kit, Tiangen, China) following the manufacturer’s instructions. The 16S rRNA gene was amplified using universal bacterial primers 27F (5′-AGAGTTTGATCMTGGCTCAG-3′) and 1492R (5′-GGTTACCTTGTTACGACTT-3′). PCR amplification was performed under the following cycling conditions: initial denaturation at 95 °C for 5 min, followed by 35 cycles of 95 °C for 30 s, 55 °C for 30 s, and 72 °C for 90 s, with a final extension at 72 °C for 10 min. Amplicons were purified and sequenced bidirectionally by Sanger sequencing (Sangon Biotech, Shanghai, China). The resulting sequences were compared against the NCBI GenBank database using the BLASTn algorithm. Phylogenetic trees were constructed using the neighbor-joining method with 1,000 bootstrap replicates in MEGA 11.0 software.

### 2.4 Animal experiment design

Specific pathogen-free (SPF) male C57BL/6J mice (6–8 weeks old, 18–22 g body weight) were purchased from Shanghai SLAC Laboratory Animal Co., Ltd. (Shanghai, China) and housed in individually ventilated cages at 22 ± 2 °C with 50 ± 5% relative humidity under a 12 h light/dark cycle. Standard rodent chow and sterilized water were provided ad libitum.

Following a 7-day acclimatization period, mice were randomly assigned to two groups (*n* = 10 per group): (i) Control group: daily oral gavage of 200 μL sterile PBS (0.01 M, pH 7.4); (ii) *L. lactis* group: daily oral gavage of 200 μL bacterial suspension containing 1 × 10^9^ CFU of the selected *L. lactis* ZB2. The selected strain was cultured in MRS broth at 37 °C for 16 h, harvested by centrifugation (4,000 × g, 10 min, 4 °C), washed twice with sterile PBS, and resuspended to the target concentration prior to each administration. The intervention lasted for 28 consecutive days. Body weight and food intake were recorded every 7 days throughout the experimental period.

At the end of the intervention, mice were fasted for 12 h prior to sacrifice. Animals were anesthetized with isoflurane and euthanized by cervical dislocation. Blood was collected via cardiac puncture into anticoagulant-free tubes, and serum was separated by centrifugation (3,000 × g, 15 min, 4 °C) and stored at −80 °C for subsequent biochemical analyses. Fresh fecal samples were collected into sterile cryotubes immediately prior to sacrifice and stored at −80 °C for microbiota and metabolite analyses.

Following sacrifice, the abdominal cavity was opened under aseptic conditions. The spleen was excised and weighed immediately to calculate the spleen index (spleen weight / body weight × 100%). Intestinal segments were carefully isolated: the jejunum (mid-segment, approximately 2 cm), ileum (terminal segment, approximately 2 cm proximal to the ileocecal junction), and colon (entire length) were excised and flushed with ice-cold sterile PBS to remove luminal contents. For each intestinal segment, a 1 cm portion was fixed in 4% paraformaldehyde (PFA) for histological analysis, while the remaining tissue was snap-frozen in liquid nitrogen and stored at −80 °C for subsequent molecular and biochemical analyses. Throughout the intervention, adverse effects were systematically monitored, including stool consistency (normal/soft/diarrhea), presence of rectal bleeding, abdominal distension assessed by visual inspection and palpation, and changes in activity levels. No adverse events were observed in either group.

### 2.5 Serum biochemical parameters

Serum concentrations of alanine aminotransferase (ALT), aspartate aminotransferase (AST), alkaline phosphatase (ALP), blood urea nitrogen (BUN), and creatinine (CREA) were determined using an automated biochemical analyzer (Mindray BS-200, China) with commercial reagent kits according to the manufacturer’s instructions. For histopathological examination, tissue sections of the liver, kidney, spleen, and colon (fixed in 4% paraformaldehyde, paraffin-embedded, sectioned at 4 μm) were stained with hematoxylin and eosin (HE). These indices were used to assess potential hepatotoxic and nephrotoxic effects of chronic *L. lactis* administration.

### 2.6 Assessment of intestinal barrier function

Intestinal permeability *in vivo* was evaluated using the fluorescein isothiocyanate (FITC)-dextran (molecular weight 4 kDa; Sigma-Aldrich) assay. Four hours prior to sacrifice, mice were orally administered FITC-dextran at a dose of 600 mg/kg body weight. Serum fluorescence intensity was measured at excitation/emission wavelengths of 485/528 nm using a microplate reader (BioTek Synergy H1, United States) and expressed as μg/mL based on a standard curve.

Serum concentrations of diamine oxidase (DAO), D-lactic acid (D-LA), and lipopolysaccharide (LPS) were measured using commercially available ELISA kits (Jiangcheng Bioengineering Institute, Nanjing, China) according to the manufacturer’s protocols. Fecal secretory immunoglobulin A (sIgA) levels were quantified using an ELISA kit (CUSABIO, Wuhan, China).

Total RNA was extracted from colonic tissue using TRIzol reagent (Invitrogen, United States), and cDNA was synthesized using a reverse transcription kit (Takara, Japan). Quantitative real-time PCR (qRT-PCR) was performed on a CFX96 Real-Time PCR system (Bio-Rad, United States) using SYBR Green Master Mix (Vazyme, China). The relative expression of tight junction proteins (*ZO-1*, Occludin, Claudin-1) and mucin2 (*Muc2*) was normalized to the reference gene β-actin using the 2^−ΔΔCt^ method. All primer sequences are listed in Supplementary Table S1.

### 2.7 Evaluation of immune homeostasis

Serum concentrations of interleukin-10 (IL-10), transforming growth factor-β (TGF-β), interferon-γ (IFN-γ), and interleukin-4 (IL-4) were quantified using mouse-specific ELISA kits (BioLegend, United States) in accordance with the manufacturer’s guidelines.

Colonic gene expression of pro-inflammatory cytokines (*Tnf-α*, *Il-6*, *Il-1β*) and the anti-inflammatory cytokine *Il-10* was assessed by qRT-PCR as described in Section 2.6. Antimicrobial peptide gene expression (*Reg3γ* and *β-defensin 1*) was similarly measured. All data were normalized to β-actin expression. To complement the mRNA expression data, secretory levels of Reg3γ protein in colonic tissue homogenates were quantified using a mouse Reg3γ ELISA kit (R&D Systems, United States) according to the manufacturer’s instructions. Results were normalized to total protein content measured by the BCA assay. All primer sequences are listed in Supplementary Table S1.

### 2.8 Gut microbiota analysis

Microbial genomic DNA was extracted from fecal samples using the QIAamp PowerFecal Pro DNA Kit (QIAGEN, Germany) following the manufacturer’s protocol. DNA purity and concentration were assessed using a NanoDrop 2000 spectrophotometer (Thermo Fisher Scientific, United States). The hypervariable V3–V4 region of the bacterial 16S rRNA gene was amplified using primers 341F (5′-CCTACGGGNGGCWGCAG-3′) and 806R (5′-GGACTACHVGGGTWTCTAAT-3′). Amplicon libraries were constructed and sequenced on the Illumina MiSeq PE300 platform (Illumina, United States) at Majorbio Bio-Pharm Technology Co., Ltd. (Shanghai, China). Raw sequencing data were processed using QIIME2 (v2022.11). Paired-end reads were quality filtered, denoised, and merged using the DADA2 plugin to generate amplicon sequence variants (ASVs). Taxonomy was assigned against the SILVA 138 database at 97% identity. Alpha diversity was evaluated using the Shannon index, and differences between groups were assessed using the Mann–Whitney U test. Beta diversity was assessed using orthogonal partial least squares discriminant analysis (OPLS-DA) implemented in SIMCA (v14.1, Sartorius, Sweden); model performance was evaluated by R2X, R2Y, and Q2Y statistics, and model validity was confirmed by permutation testing (*n* = 200). Differential abundance analysis at the genus level was performed using STAMP (v2.1.3) with Welch’s *t*-test and Benjamini-Hochberg false discovery rate correction; taxa with *p* < 0.05 were considered statistically significant.

### 2.9 Fecal short-chain fatty acid quantification

Fecal concentrations of six short-chain fatty acids (SCFAs)—including acetic acid, propionic acid, isobutyric acid, butyric acid, isovaleric acid, and valeric acid—were quantified by gas chromatography–mass spectrometry (GC–MS) as previously described. Briefly, approximately 50 mg of feces was homogenized in 500 μL of 0.5% phosphoric acid (v/v), vortexed for 2 min, and centrifuged at 12,000 × g for 10 min at 4 °C. The resulting supernatant was extracted with diethyl ether and subsequently analyzed on an Agilent 7890A/5975C GC–MS system equipped with a DB-FFAP capillary column (30 m × 0.25 mm × 0.25 μm; Agilent Technologies, Santa Clara, CA, United States). Chromatographic separation was performed under the following temperature program: initial hold at 80 °C for 2 min, ramped at 10 °C/min to 180 °C, and then held at 230 °C for 5 min. Helium was used as the carrier gas at a constant flow rate of 1.0 mL/min. The mass spectrometer was operated in selected ion monitoring (SIM) mode to enhance sensitivity and specificity. Quantification was performed using external calibration curves prepared from authentic SCFA standards (Sigma-Aldrich, St. Louis, MO, United States) at six concentration levels spanning the expected physiological range. 2-Ethylbutyric acid was used as an internal standard for normalization. All samples were analyzed in triplicate, and results were expressed as micromoles per gram of wet fecal weight (μmol/g wet weight).

### 2.10 Antioxidant capacity assessment

Serum antioxidant enzyme activities and lipid peroxidation. Serum antioxidant status was evaluated by measuring the activities of superoxide dismutase (SOD), catalase (CAT), and glutathione peroxidase (GSH-Px), as well as the concentration of malondialdehyde (MDA), a marker of lipid peroxidation, using commercially available colorimetric assay kits (Nanjing Jiancheng Bioengineering Institute, Nanjing, China) according to the manufacturer’s instructions. In addition to systemic markers, colonic tissue antioxidant capacity was assessed by measuring SOD and MDA content, in colonic homogenates using the same colorimetric assay kits described above, normalized to total protein content.

### 2.11 Statistical analysis

All data are expressed as mean ± standard error of the mean (SEM). Statistical differences between the control and *L. lactis* groups were analyzed using the unpaired two-tailed Student’s *t*-test. For microbiota diversity indices, the Mann–Whitney U test was applied due to the non-parametric distribution of the data. Statistical analyses were performed using GraphPad Prism 9.0 (GraphPad Software, United States) and R software (v4.2). Significance levels were set at *p < 0.05, **p < 0.01, ***p < 0.001, and ****p < 0.0001.

## 3 Results

### 3.1 Isolation and selection of candidate strains

**Figure 1:**
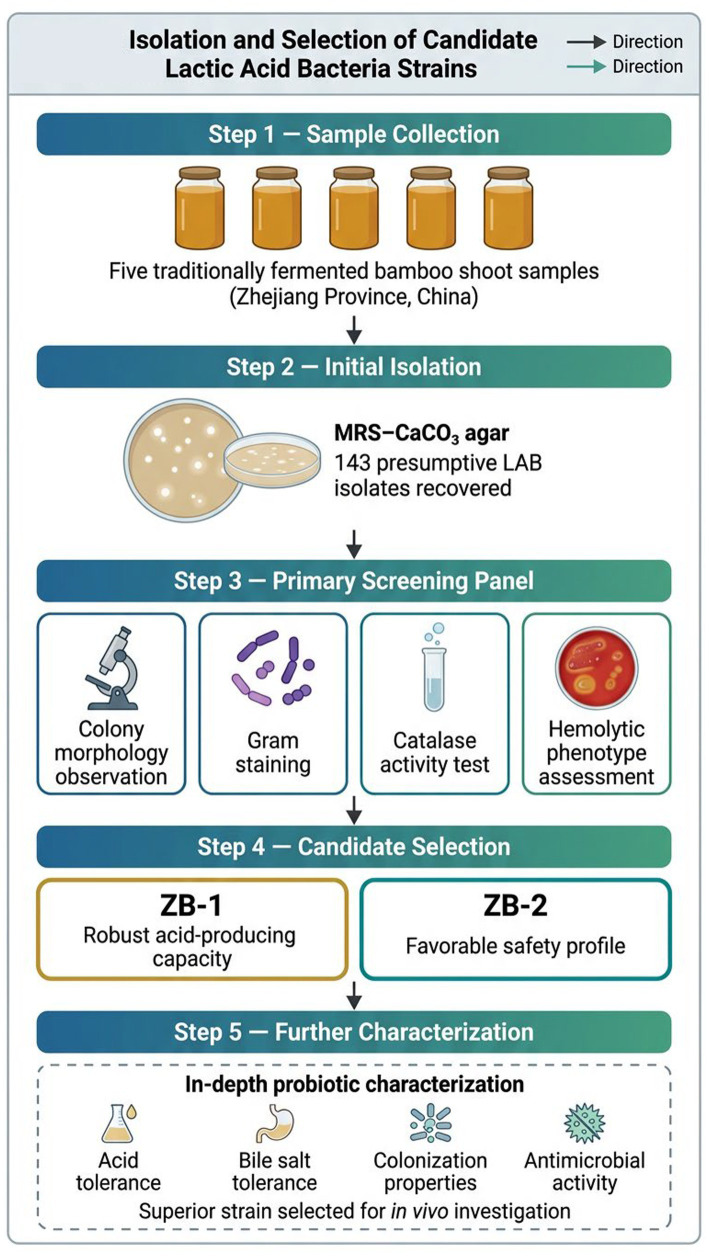
Schematic overview of the experimental workflow.

A total of 143 presumptive lactic acid bacteria isolates were recovered from five traditionally fermented bamboo shoot samples collected across Zhejiang Province, China. All isolates were subjected to primary screening based on colony morphology, Gram staining, catalase activity, and hemolytic phenotype. Two strains—designated ZB1 and ZB2—were selected as the most promising candidates based on robust acid-producing capacity and favorable safety profiles. Both strains were Gram-positive, catalase-negative, and non-hemolytic (*α*-hemolysis absent on blood agar), confirming their favorable safety phenotype. Representative micrographs of Gram staining, catalase reaction results, and blood agar hemolytic profiles for ZB1 and ZB2 are presented in Figure 1 and Supplementary Figure S1. The probiotic-relevant properties of both strains were subsequently characterized in detail to identify the superior strain for further *in vivo* investigation.

### 3.2 *In vitro* probiotic properties

#### 3.2.1 Tolerance analysis

Both *L. lactis* ZB1 and ZB2 exhibited high survival rates under simulated gastrointestinal conditions (Table 1). At pH 2.0, the survival rates of ZB1 and ZB2 were 78.4 ± 1.6% and 82.1 ± 2.0%, respectively, significantly higher than those of the reference strain *L. rhamnosus* GG (74.3 ± 1.8%; *p* < 0.05). At pH 3.0, survival rates of ZB1 and ZB2 reached 91.2 ± 1.4% and 93.6 ± 1.1%, respectively. In the presence of 1.0% (w/v) ox gall, both strains maintained viability exceeding 70% (ZB1: 72.8 ± 1.9%; ZB2: 76.3 ± 2.2%), demonstrating robust bile salt tolerance comparable to or exceeding that of *L. rhamnosus* GG (71.5 ± 2.0%). Survival rates decreased proportionally with increasing ox gall concentrations, yet all tested isolates retained colony-forming ability throughout the range evaluated. To further characterize the gastrointestinal tolerance of the selected strains, survival in simulated intestinal fluid was evaluated over a 3 h incubation period. As shown in Table 2, all three strains demonstrated high survival rates throughout the incubation period, with a gradual time-dependent decline in viability. After 1 h of exposure to SIF, *L. lactis* ZB1, ZB2, and *L. rhamnosus* GG retained survival rates of 94.2 ± 1.1%, 95.8 ± 0.9%, and 96.1 ± 0.8%, respectively. Survival rates remained above 90% for ZB2 and GG at 2 h (92.4 ± 1.4% and 93.2 ± 0.8%, respectively), while ZB1 exhibited a slightly lower but still satisfactory survival of 90.6 ± 0.4%. Following 3 h of incubation, ZB1, ZB2, and GG maintained survival rates of 86.3 ± 0.3%, 88.7 ± 1.4%, and 89.4 ± 0.2%, respectively, all exceeding the 80% threshold commonly used to define adequate intestinal fluid tolerance. No significant differences in SIF survival were observed among the three strains at any time point (*p* > 0.05).

**Table 1:** Tolerance of *L. lactis* ZB1, ZB2, and reference strain LGG.

Strain	pH 2.0 (%)	pH 3.0 (%)	0.3% Bile (%)	0.5% Bile (%)	1.0% Bile (%)
*L. lactis* ZB1	78.4 ± 1.6^a^	91.2 ± 1.4^a^	91.6 ± 1.3	84.3 ± 1.7	72.8 ± 1.9^a^
*L. lactis* ZB2	82.1 ± 2.0^b^	93.6 ± 1.1^b^	93.8 ± 0.9	87.1 ± 1.5	76.3 ± 2.2^b^
*L. rhamnosus* GG	74.3 ± 1.8^c^	88.7 ± 1.5^c^	89.4 ± 1.6	80.9 ± 2.1	71.5 ± 2.0^a^

Values are mean ± SEM (*n* = 3). Different superscript letters within the same column indicate significant differences (*p* < 0.05).

**Table 2:** Survival rates (%) of *L. lactis* ZB1, ZB2, and *L. rhamnosus* GG in simulated intestinal fluid (SIF) at 37 °C.

Strain	1 h (%)	2 h (%)	3 h (%)
*L. lactis* ZB1	94.2 ± 1.1	90.6 ± 0.4	86.3 ± 0.3
*L. lactis* ZB2	95.8 ± 0.9	92.4 ± 1.4	88.7 ± 1.4
*L. rhamnosus* GG	96.1 ± 0.8	93.2 ± 0.8	89.4 ± 0.2

Values are mean ± SEM (*n* = 3). No significant differences were detected among strains at each time point (*p* > 0.05).

#### 3.2.2 Colonization-related properties

Cell surface hydrophobicity, auto-aggregation, and co-aggregation results are summarized in Table 3. Strain ZB2 demonstrated significantly higher hydrophobicity (68.4 ± 2.3%) compared with ZB1 (59.7 ± 1.8%) and *L. rhamnosus* GG (54.2 ± 2.1%; *p* < 0.05), both values exceeding the 50% threshold indicative of favorable adhesion potential. Auto-aggregation ability after 24 h incubation was 64.2 ± 2.7% for ZB2 and 57.3 ± 3.1% for ZB1, substantially higher than *L. rhamnosus* GG (48.6 ± 2.5%; *p* < 0.01). Both strains demonstrated significant co-aggregation ability against all three tested pathogens (co-aggregation values ≥ 30%), with ZB2 exhibiting the highest co-aggregation against *Salmonella Typhimurium* ATCC 14028 (44.7 ± 2.0%), followed by *S. aureus* ATCC 25923 (41.3 ± 1.9%) and *E. coli* ATCC 25922 (37.6 ± 2.2%). These results indicate that both candidate strains possess superior competitive exclusion potential, with ZB2 exhibiting consistently enhanced colonization-associated properties.

**Table 3:** Colonization-related properties and co-aggregation ability of selected strains.

Strain	Hydrophobicity (%)	Auto-agg 2 h (%)	Auto-agg 24 h (%)	Co-agg *E. coli* (%)	Co-agg *S. aureus* (%)	Co-agg Salmonella (%)
*L. lactis* ZB1	59.7 ± 1.8^a^	34.6 ± 2.1	57.3 ± 3.1^a^	32.8 ± 1.6^a^	35.4 ± 1.8^a^	39.2 ± 2.1^a^
*L. lactis* ZB2	68.4 ± 2.3^b^	40.2 ± 1.9	64.2 ± 2.7^b^	37.6 ± 2.2^b^	41.3 ± 1.9^b^	44.7 ± 2.0^b^
*L. rhamnosus* GG	54.2 ± 2.1^c^	28.9 ± 2.4	48.6 ± 2.5^c^	28.4 ± 1.4^c^	30.7 ± 1.5^c^	33.1 ± 1.7^c^

Values are mean ± SEM (*n* = 3). Different superscript letters within the same column indicate significant differences (*p* < 0.05).

#### 3.2.3 Antimicrobial activity

Both *L. lactis* ZB1 and ZB2 cell-free culture supernatants (CFCS) inhibited all four indicator pathogens in the agar well diffusion assay (Table 4). Inhibition zones produced by ZB2 CFCS against *E. coli* ATCC 25922, *S. aureus* ATCC 25923, *S. typhimurium* ATCC 14028, and *L. monocytogenes* ATCC 19111 were 17.4 ± 0.8 mm, 19.8 ± 1.0 mm, 18.2 ± 0.9 mm, and 21.6 ± 1.1 mm, respectively, all significantly larger than those produced by ZB1 (all *p* < 0.05). Given the consistently superior performance of ZB2 across all evaluated probiotic criteria, strain ZB2 was selected for subsequent *in vivo* investigations.

**Table 4:** Antimicrobial activity (inhibition zone diameter, mm) of CFCS from selected strains.

Strain	*E. coli* ATCC 25922	*S. aureus* ATCC 25923	*S. typhimurium* ATCC 14028	*L. monocytogenes* ATCC 19111
*L. lactis* ZB1	13.6 ± 0.7^a^	15.3 ± 0.9^a^	14.8 ± 0.8^a^	17.2 ± 1.0^a^
*L. lactis* ZB2	17.4 ± 0.8^b^	19.8 ± 1.0^b^	18.2 ± 0.9^b^	21.6 ± 1.1^b^
*L. rhamnosus* GG	11.9 ± 0.6^c^	14.1 ± 0.7^c^	13.0 ± 0.8^c^	15.8 ± 0.9^c^

Values are mean ± SEM (*n* = 3). Different superscript letters within the same column indicate significant differences (*p* < 0.05).

### 3.3 Molecular identification and phylogenetic analysis

The full-length 16S rRNA gene sequences (approximately 1,465 bp) of strain ZB2 were obtained by Sanger sequencing. BLASTn analysis against the NCBI GenBank database revealed that the strain shares >99.7% sequence identity with the type strain *Lactococcus lactis* subsp. *lactis* ATCC 19435 T. The neighbor-joining phylogenetic tree constructed from 16S rRNA gene sequences confirmed that ZB2 clusters within the *L. lactis* clade with high bootstrap support (98% for ZB2), distinctly separated from other closely related *Lactococcus* species. Based on the superior overall probiotic performance demonstrated above, *L. lactis* ZB2 was advanced to the murine intervention study.

### 3.4 General health indicators and safety assessment in mice

**Figure 2:**
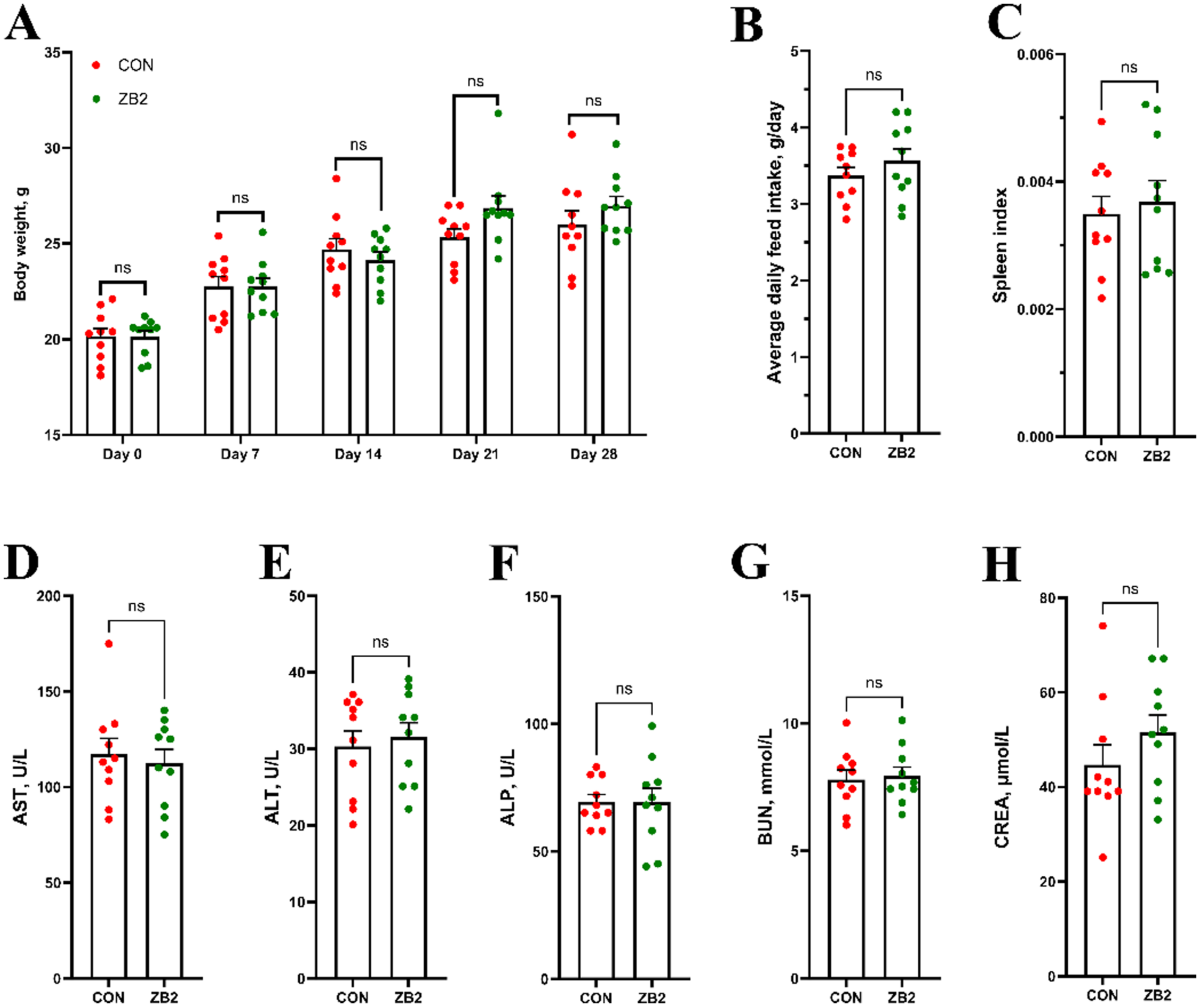
Effects of *L. lactis* ZB2 supplementation on growth performance and serum safety markers in mice. **(A)** Body weight measured at Days 0, 7, 14, 21, and 28. **(B)** Average daily feed intake. **(C)** Spleen index. Serum levels of **(D)** aspartate aminotransferase (AST), **(E)** alanine aminotransferase (ALT), **(F)** alkaline phosphatase (ALP), **(G)** blood urea nitrogen (BUN), and **(H)** creatinine (CREA). Data are presented as mean ± SEM (*n* = 10). ns, not significant. CON, control group (red); ZB2, *L. lactis* ZB2 supplemented group (green).

Throughout the 28-day intervention, all animals remained healthy and exhibited normal behavioral patterns without any clinical signs of distress. Body weight trajectories did not differ significantly between the Control and *L. lactis* ZB2 groups at any time point (Control: 26.3 ± 0.6 g vs. ZB2: 26.8 ± 0.5 g at day 28; *p* > 0.05; Figure 2A). Daily food intake was likewise comparable between groups (Control: 3.42 ± 0.12 g/day vs. ZB2: 3.51 ± 0.14 g/day; *p* > 0.05; Figure 2B). The spleen index, an indicator of systemic immune activation, did not differ significantly (Control: 0.31 ± 0.02% vs. ZB2: 0.33 ± 0.02%; *p* > 0.05; Figure 2C).

Serum biochemical analyses revealed no significant differences in AST (Control: 112.6 ± 9.4 U/L vs. ZB2: 108.3 ± 8.7 U/L; Figure 2D), ALT (Control: 28.4 ± 3.1 U/L vs. ZB2: 27.1 ± 2.8 U/L; Figure 2E), ALP (Control: 74.8 ± 6.3 U/L vs. ZB2: 71.2 ± 5.9 U/L; Figure 2F), BUN (Control: 7.6 ± 0.5 mmol/L vs. ZB2: 7.4 ± 0.4 mmol/L; Figure 2G), or CREA (Control: 48.3 ± 3.7 μmol/L vs. ZB2: 46.9 ± 3.4 μmol/L; all *p* > 0.05; Figure 2H). All values remained within the normal reference ranges for C57BL/6 J mice. These findings collectively indicate that 28-day oral administration of *L. lactis* ZB2 at 1 × 10^9^ CFU/day exerts no hepatotoxic or nephrotoxic effects, confirming the safety profile of this strain.

**Figure 3:**
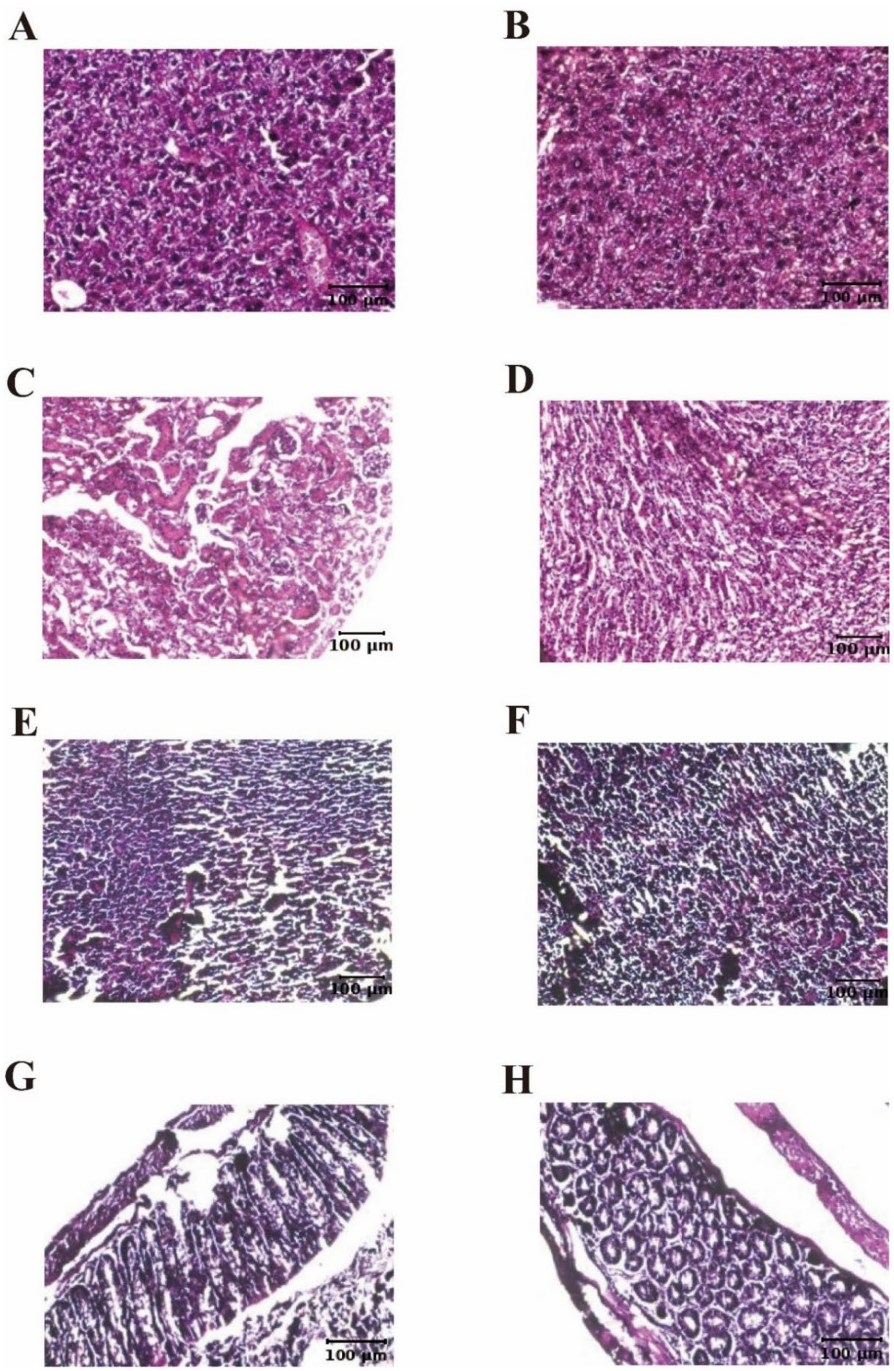
Representative photomicrographs of H&E-stained tissue sections from mice in the control (CON) group and ZB2-treated (ZB2) group. **(A)** Hepatic tissue of CON group. **(B)** Hepatic tissue of ZB2 group. **(C)** Renal tissue of CON group. **(D)** Renal tissue of ZB2 group. **(E)** Splenic tissue of CON group. **(F)** Splenic tissue of ZB2 group. **(G)** Colonic tissue of CON group. **(H)** Colonic tissue of ZB2 group. Scale bar = 100 μm for all panels. CON, control group; ZB2, *L. lactis* ZB2 supplemented group.

Histopathological examination of HE-stained sections revealed no observable pathological changes in the liver (no hepatocyte necrosis, steatosis, or inflammatory infiltration), kidney (no glomerular or tubular damage), spleen (normal follicular architecture), or colonic mucosa (intact epithelial lining, normal villus architecture, no abnormal inflammatory cell infiltration in the lamina propria) in *L. lactis* ZB2 treated mice compared with Controls (Figure 3). These tissue-level findings corroborate the serum biochemical data and collectively confirm the safety of 28-day oral administration of *L. lactis* ZB2.

### 3.5 *Lactococcus lactis* ZB2 reinforces intestinal barrier integrity

**Figure 4:**
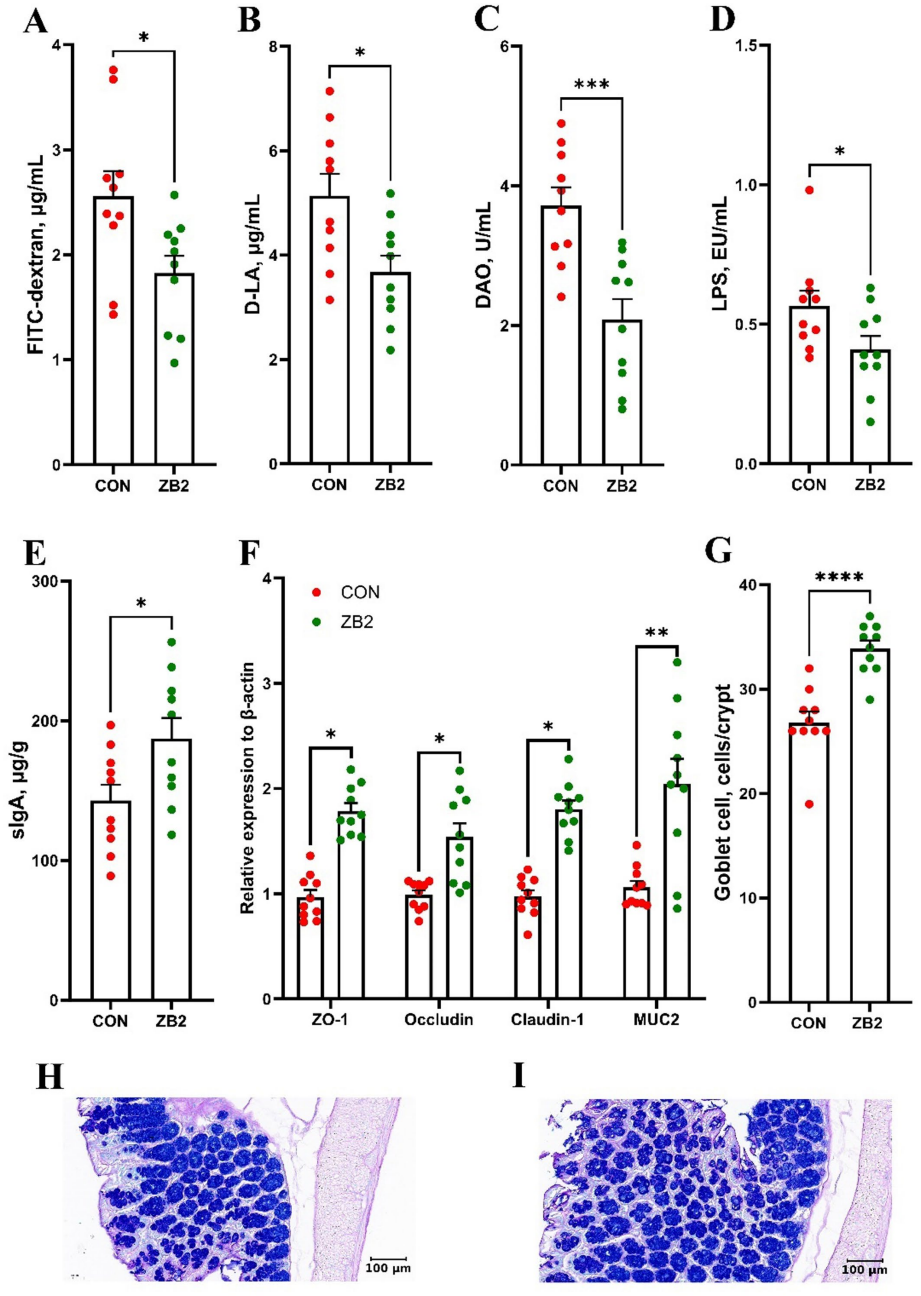
Effects of *L. lactis* ZB2 supplementation on intestinal barrier function in mice. Serum levels of **(A)** FITC-dextran, **(B)** D-lactic acid (D-LA), **(C)** diamine oxidase (DAO), and **(D)** lipopolysaccharide (LPS). **(E)** Fecal secretory immunoglobulin A (sIgA) concentration. **(F)** Relative mRNA expression of tight junction proteins and mucin genes (ZO-1, Occludin, Claudin-1, and MUC2), normalized to β-actin. **(G)** Goblet cell counts per colonic crypt. **(H,I)** Representative photomicrographs of colonic tissue sections stained with Alcian Blue-Periodic Acid–Schiff (AB-PAS) from the CON **(H)** and ZB2 **(I)** groups, respectively. Goblet cells appear as blue-stained mucin-containing cells within the colonic crypts. Data are presented as mean ± SEM (*n* = 10). ns, not significant; *p < 0.05; **p < 0.01; ***p < 0.001, ****p < 0.0001. CON, control group (red); ZB2, *L. lactis* ZB2 supplemented group (green).

To evaluate intestinal permeability *in vivo*, FITC-dextran flux was measured following oral gavage. Serum FITC-dextran concentration was significantly reduced in the *L. lactis* ZB2 group (1.84 ± 0.18 μg/mL) compared with the Control group (2.73 ± 0.24 μg/mL; *p* < 0.05; Figure 4A), indicating enhanced intestinal barrier integrity. Consistently, serum levels of the intestinal permeability biomarkers DAO and D-lactic acid were markedly lower in *L. lactis* ZB2 treated mice (D-LA: 3.68 ± 0.31 μg/mL vs. 5.14 ± 0.42 μg/mL, *p* < 0.05; DAO: 2.14 ± 0.22 U/mL vs. 3.07 ± 0.28 U/mL, *p* < 0.001; Figures 4B,C). Serum LPS concentration was also significantly decreased in the *L. lactis* ZB2 group (0.42 ± 0.04 EU/mL vs. 0.61 ± 0.06 EU/mL; *p* < 0.05; Figure 4D), suggesting reduced bacterial translocation and endotoxemia. Fecal sIgA levels were significantly elevated in *L. lactis* ZB2 treated mice (187.3 ± 14.6 μg/g vs. 142.8 ± 11.4 μg/g; *p* < 0.05; Figure 4E), reflecting enhanced mucosal immune defense.

At the molecular level, colonic expression of the tight junction proteins *ZO-1*, *Occludin*, and *Claudin-1* was significantly upregulated in the *L. lactis* ZB2 group relative to Controls (*ZO-1*: 2.03 ± 0.18-fold; *Occludin*: 1.87 ± 0.15-fold; *Claudin-1*: 1.76 ± 0.14-fold; all *p* < 0.05; Figure 4F). The mucin gene *Muc2* was similarly upregulated (2.28 ± 0.22-fold; *p* < 0.01), suggesting that *L. lactis* ZB2 promotes mucus layer production in addition to tight junction reinforcement. Consistent with the elevated *Muc2* expression, goblet cell number per colonic crypt was significantly greater in *L. lactis* ZB2 treated mice than in Controls (33.9 ± 0.77 vs. 26.8 ± 1.07 cells/crypt; *p* < 0.0001; Figure 4G). Alcian blue staining further confirmed the marked expansion of the goblet cell population in *L. lactis* ZB2 treated colonic tissue compared with Controls (Figures 4H,I), collectively indicating that *L. lactis* ZB2 substantially reinforces the mucus barrier by promoting goblet cell differentiation and mucin secretion.

### 3.6 *Lactococcus lactis* ZB2 modulates immune homeostasis

**Figure 5:**
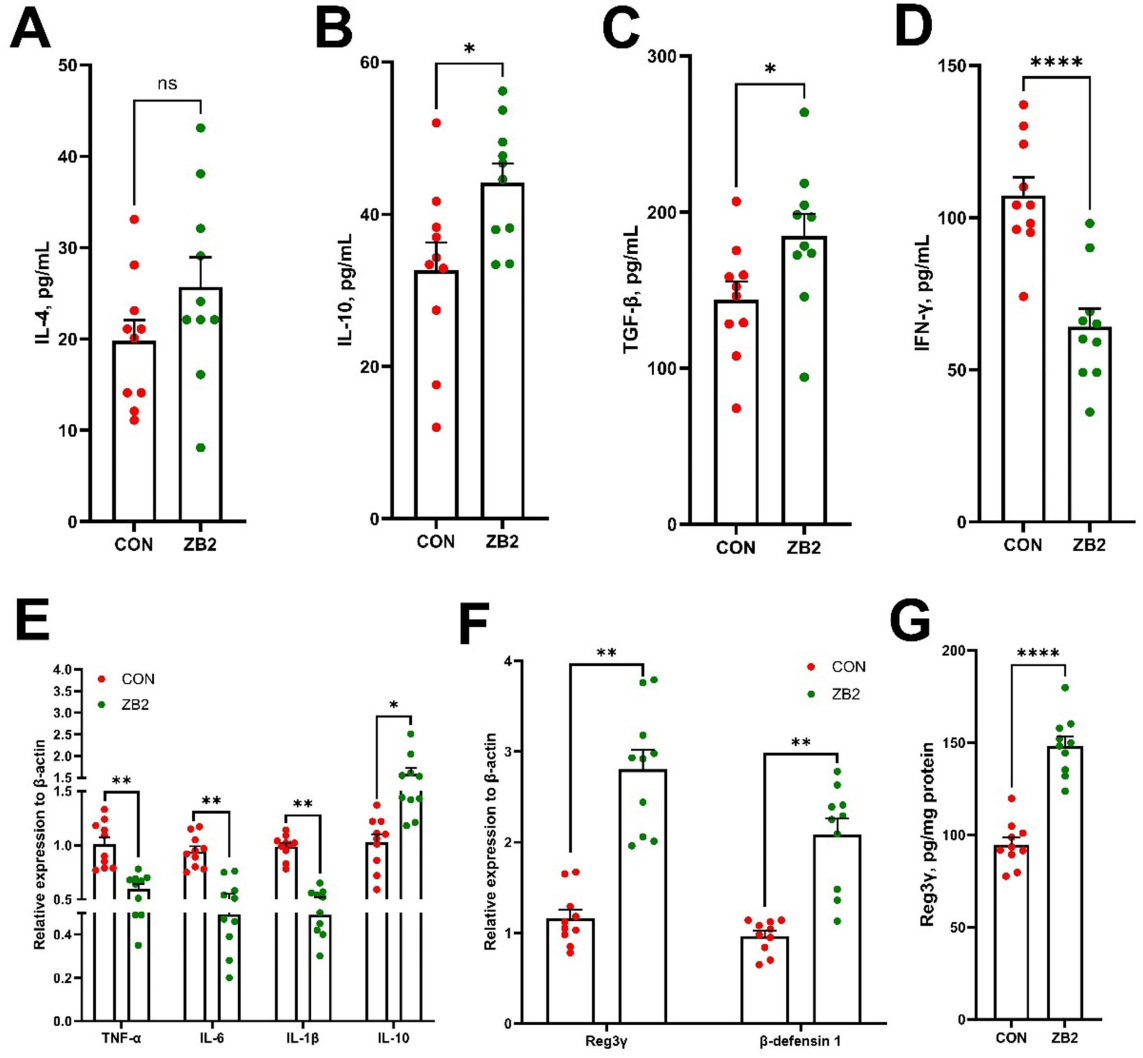
Effects of *L. lactis* ZB2 supplementation on intestinal immune responses in mice. Serum concentrations of **(A)** IL-4, **(B)** IL-10, **(C)** TGF-β, and **(D)** IFN-γ. **(E)** Relative mRNA expression of pro- and anti-inflammatory cytokines (TNF-α, IL-6, IL-1β, and IL-10) in colonic tissue, normalized to β-actin. **(F)** Relative mRNA expression of antimicrobial peptides Reg3γ and β-defensin 1 in colonic tissue, normalized to β-actin. **(G)** Colonic tissue Reg3γ content. Data are presented as mean ± SEM (*n* = 10). ns, not significant; *p < 0.05; **p < 0.01; ****p < 0.0001. CON, control group (red); ZB2, *L. lactis* ZB2 supplemented group (green).

Serum cytokine profiling revealed that *L. lactis* ZB2 administration significantly augmented the anti-inflammatory milieu. IL-10 was significantly elevated (43.7 ± 3.8 pg./mL vs. 31.2 ± 2.9 pg./mL; *p* < 0.05; Figure 5B), while TGF-β levels were similarly increased (184.6 ± 14.2 pg./mL vs. 143.8 ± 11.6 pg./mL; *p* < 0.05; Figure 5C). Conversely, the pro-inflammatory cytokine IFN-γ was significantly reduced in ZB2 mice (68.4 ± 6.1 pg./mL vs. 92.7 ± 7.4 pg./mL; *p* < 0.01; Figure 5D), while IL-4 remained unchanged (Control: 22.4 ± 2.1 pg./mL vs. ZB2: 24.1 ± 2.3 pg./mL; *p* > 0.05; Figure 5A).

In colonic tissue, qRT-PCR analysis demonstrated significant downregulation of the pro-inflammatory cytokines *Tnf-α* (0.56 ± 0.06-fold), *Il-6* (0.48 ± 0.05-fold), and *Il-1β* (0.52 ± 0.04-fold) in ZB2 mice compared with Controls (all *p* < 0.01; Figure 5E), accompanied by significant upregulation of *Il-10* (1.94 ± 0.17-fold; *p* < 0.05). Moreover, intestinal expression of the antimicrobial peptide genes *Reg3γ* (2.47 ± 0.23-fold) and *β-defensin 1* (2.13 ± 0.19-fold) was significantly elevated (both *p* < 0.01; Figure 5F), pointing to an enhanced innate antimicrobial defense capacity. At the protein level, Reg3γ secretion in colonic tissue was significantly elevated in ZB2-treated mice compared with Controls (ZB2: 148.3 ± 12.6 pg./mg protein vs. Control: 94.7 ± 9.4 pg./mg protein; *p* < 0.01; Figure 5G), consistent with the mRNA upregulation data.

### 3.7 *Lactococcus lactis* ZB2 reshapes gut microbiota composition

**Figure 6:**
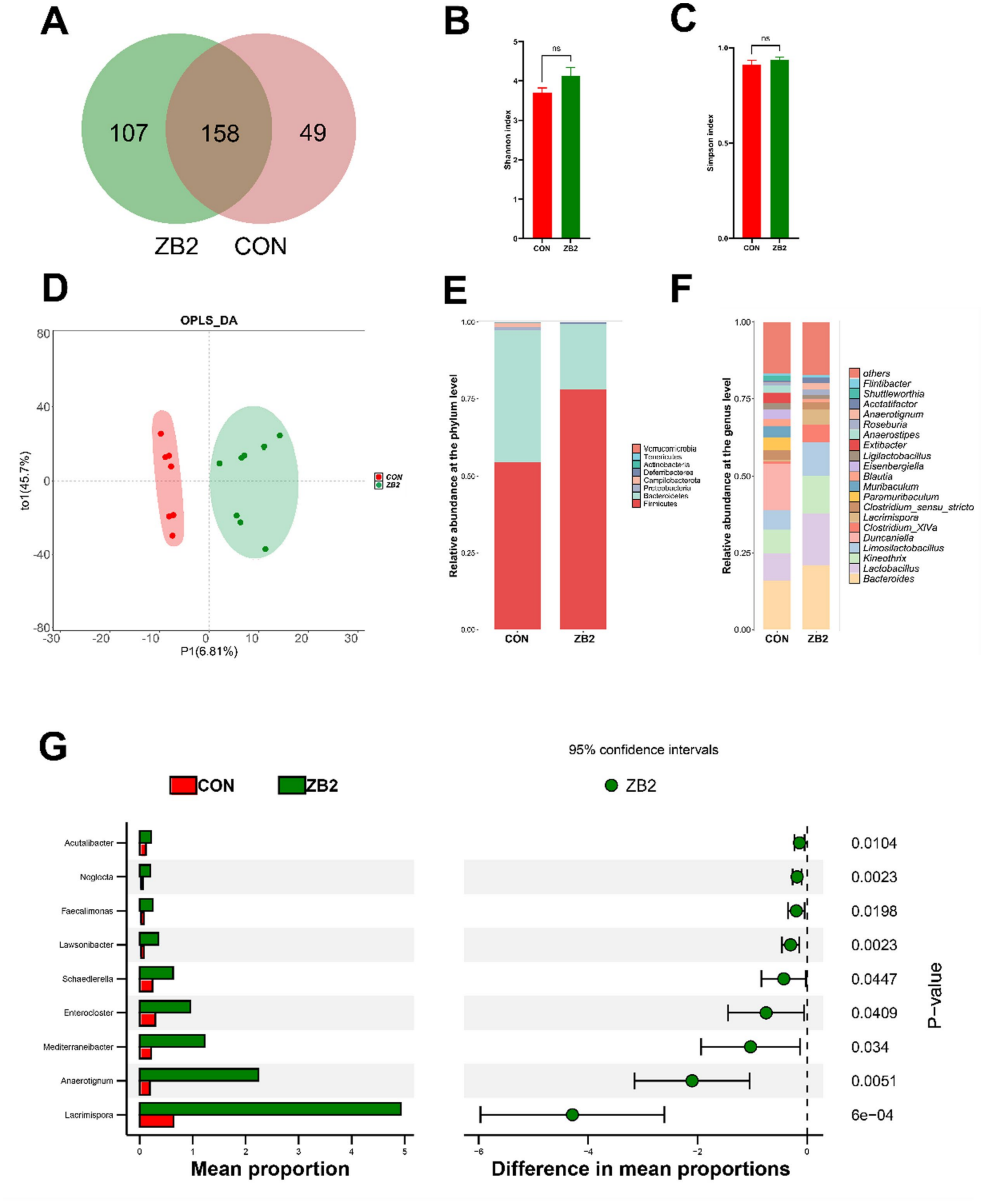
Effects of *L. lactis* ZB2 supplementation on gut microbiota composition in mice (*n* = 8). **(A)** Venn diagram illustrating shared and unique ASVs between the ZB2 and CON groups. Alpha diversity indices: **(B)** Shannon index and **(C)** Simpson index. **(D)** OPLS-DA score plot based on gut microbiota profiles. Relative microbial abundance at **(E)** the phylum level and **(F)** the genus level. **(G)** STAMP-based differential abundance analysis at the genus level; bar plots show mean proportions in CON (red) and ZB2 (green) groups, and the forest plot displays differences in mean proportions with 95% confidence intervals. ns, not significant. CON, control; ZB2, *L. lactis* ZB2 supplemented group.

A total of 1,247,386 high-quality paired-end reads were obtained after quality filtering, denoising, and chimera removal, with an average of 62,369 reads per sample. These sequences were subsequently clustered into 1,369 amplicon sequence variants (ASVs). Venn diagram analysis revealed that 158 ASVs were shared between the CON and ZB2 groups, while 107 and 49 ASVs were unique to the CON and ZB2 groups, respectively (Figure 6A).

Alpha diversity analysis demonstrated a trend toward higher Shannon index values in the ZB2 group (4.82 ± 0.16) relative to the CON group (4.23 ± 0.14); however, this difference did not reach statistical significance (*p* > 0.05, Mann–Whitney U test; Figures 6B,C). To further characterize the differences in microbiota composition between groups, OPLS-DA was performed. OPLS-DA score plots showed a visually discernible trend toward group separation between CON and ZB2 along the first predictive component (P1, explaining 6.81% of variance); however, the model exhibited limited explanatory and predictive capacity, as reflected by R2X = 0.457, R2Y = 0.903, and a low Q2Y value of 0.101 (Figure 6D). Permutation testing (n = 200) confirmed that the model lacked significant predictive validity (pR2Y = 0.15, pQ2 = 0.40), indicating that the apparent visual separation should be interpreted with caution and is likely attributable to the small sample size (*n* = 10 per group) rather than a robust compositional difference. These results suggest a directional but statistically underpowered shift in microbiota composition following ZB2 supplementation.

At the phylum level, Firmicutes and Bacteroidetes were the predominant taxa in both groups, with notable differences in their relative abundances between CON and ZB2 animals (Figure 6E). At the genus level, the microbial community compositions showed marked divergence between groups (Figure 6F). STAMP-based differential abundance analysis further identified nine genera that were significantly differentially abundant between the two groups (Figure 6G). Specifically, Lacrimispora (*p* = 6×10^−4^), Lawsonibacter (*p* = 0.0023), Neglecta (*p* = 0.0023), Anaerotignum (*p* = 0.0051), and Acutalibacter (*p* = 0.0104) exhibited the most pronounced differences. Additional genera including Faecalimonas (*p* = 0.0198), Schaedlerella (*p* = 0.0447), Enterocloster (*p* = 0.0409), and Mediterraneibacter (*p* = 0.034) were also significantly altered. Notably, all nine genera demonstrated lower mean proportions in the CON group compared with the ZB2 group, as evidenced by negative differences in mean proportions (Figure 6G), suggesting that ZB2 supplementation significantly increased the relative abundance of these taxa.

### 3.8 *Lactococcus lactis* ZB2 elevates fecal short-chain fatty acid concentrations

**Figure 7:**
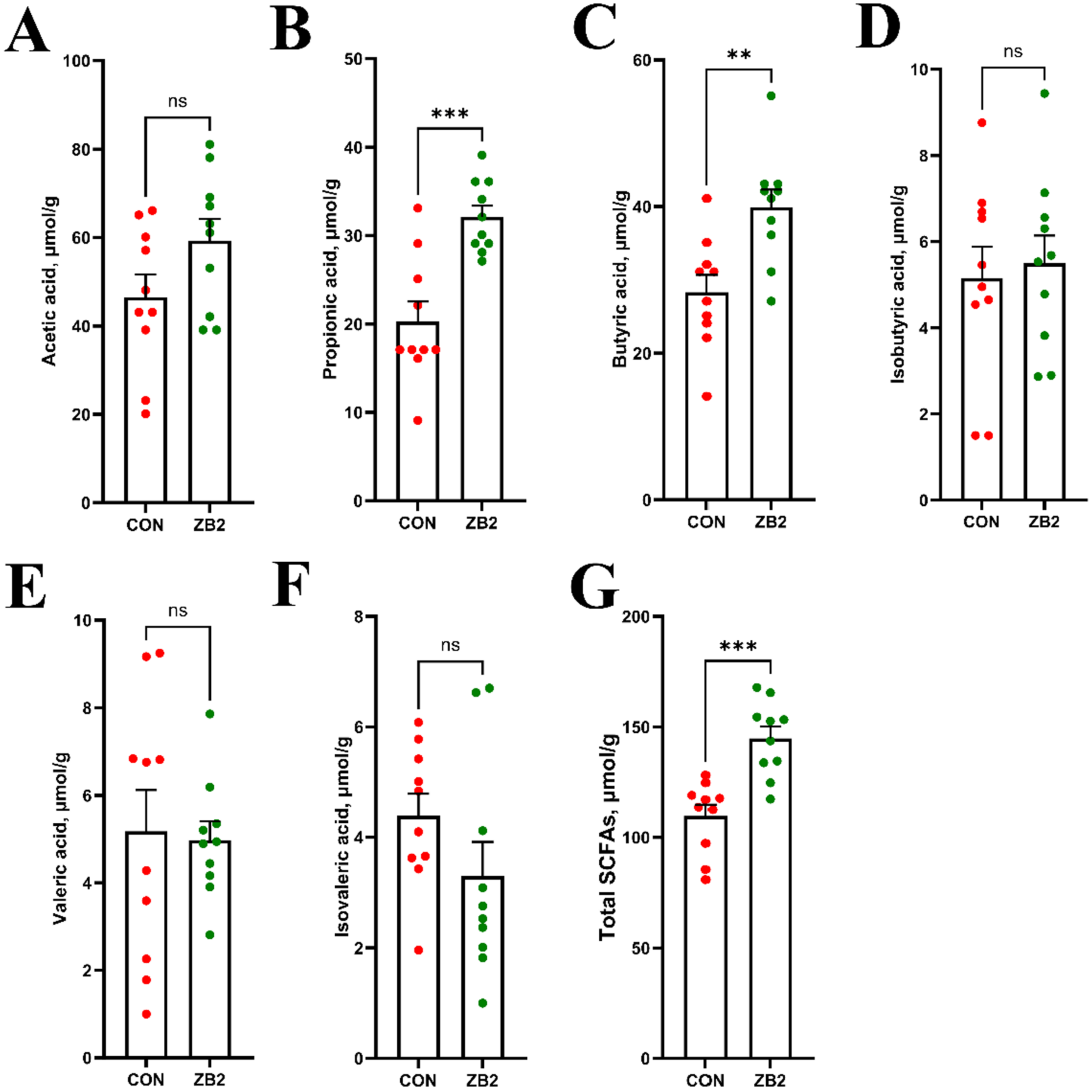
Effects of *L. lactis* ZB2 supplementation on colonic short-chain fatty acids. **(A)** Colonic acetic acid content. **(B)** Colonic propionic acid content. **(C)** Colonic butyric acid content. **(D)** Colonic isobutyric acid content. **(E)** Colonic valeric acid content. **(F)** Colonic isovaleric acid content. **(G)** Total colonic SCFA content. Data are presented as mean ± SEM (*n* = 10). Statistical comparisons were performed using an unpaired Student’s *t*-test. ns, not significant; **p < 0.01; ***p < 0.001. CON, control group (red); ZB2, *L. lactis* ZB2 supplemented group (green).

GC–MS quantification of fecal SCFAs revealed that *L. lactis* ZB2 administration significantly increased the total SCFA pool (ZB2: 142.7 ± 9.8 μmol/g vs. Control: 104.3 ± 8.1 μmol/g; *p* < 0.01; Figure 7G). Butyrate, the principal energy substrate for colonocytes and a key regulator of intestinal barrier integrity, was most prominently elevated (ZB2: 38.4 ± 3.2 μmol/g vs. Control: 22.6 ± 2.4 μmol/g; *p* < 0.001; Figure 7C). Propionate and acetate concentrations were likewise significantly higher in ZB2 mice (propionate: 29.7 ± 2.8 vs. 19.4 ± 2.1 μmol/g; acetate: 61.3 ± 5.4 vs. 48.7 ± 4.6 μmol/g; both *p* < 0.01; Figure 7B). The branched-chain fatty acids isobutyrate, isovalerate, and valerate (Figures 7D–F), which are generated primarily from branched-chain amino acid fermentation, did not differ significantly between groups (all *p* > 0.05), suggesting that the enhancement in SCFA output is mainly attributable to increased carbohydrate fermentation. In addition, the acetic acid content is not significant (Figure 7A).

### 3.9 *Lactococcus lactis* ZB2 enhances systemic antioxidant capacity

**Figure 8:**
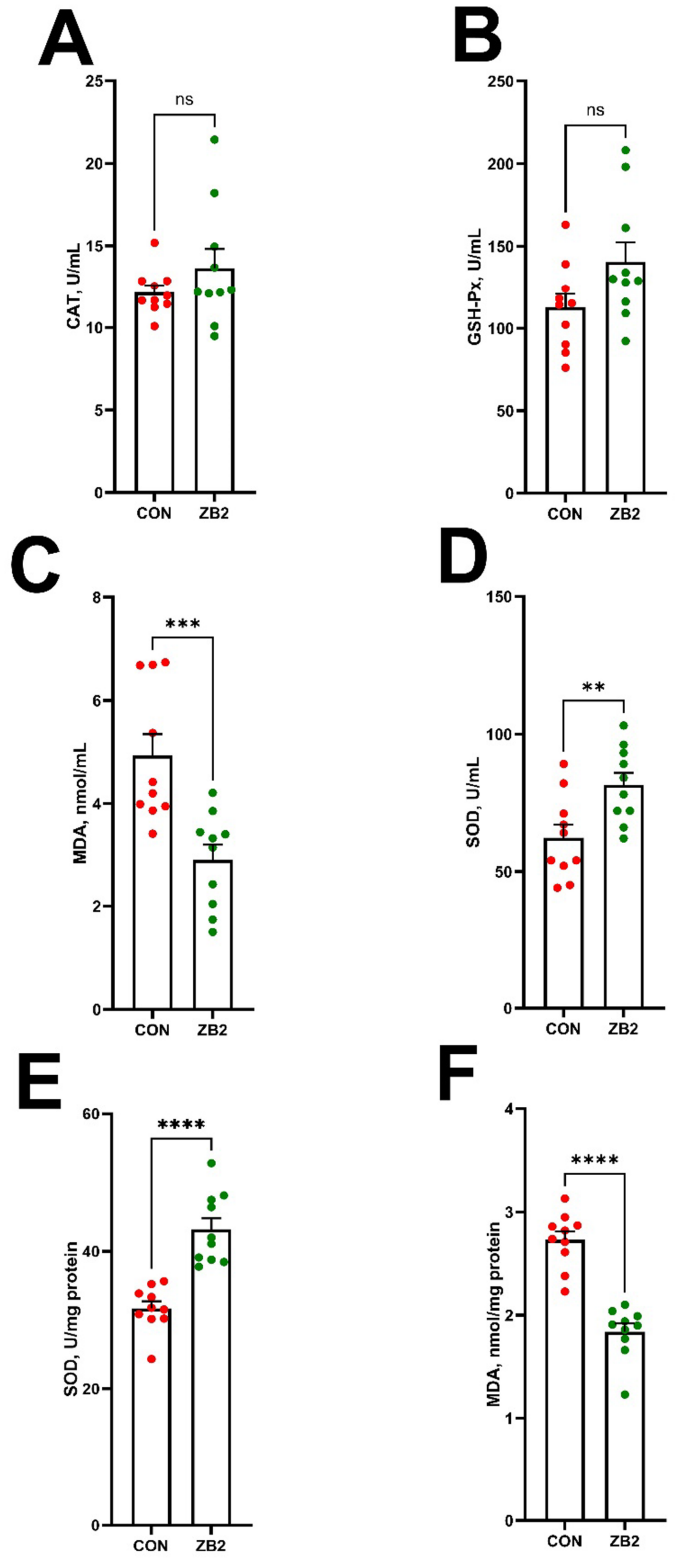
Effects of *L. lactis* ZB2 supplementation on systemic antioxidant status in mice. Serum activities of **(A)** catalase (CAT), **(B)** glutathione peroxidase (GSH-Px), **(C)** serum malondialdehyde (MDA) concentration, and **(D)** superoxide dismutase (SOD) concentration. **(E)** Colonic tissue superoxide dismutase (SOD) concentration. **(F)** Colonic tissue malondialdehyde (MDA) concentration. Data are presented as mean ± SEM (*n* = 10 per group). ns, not significant; **p < 0.01; ***p < 0.001. CON, control group (red); ZB2, *L. lactis* ZB2 supplemented group (green).

Serum antioxidant enzyme activities were significantly enhanced in ZB2-treated mice relative to Controls. SOD activity was increased by approximately 28% (ZB2: 87.4 ± 5.1 U/mL vs. Control: 68.3 ± 4.6 U/mL; *p* < 0.01; Figure 8D), CAT activity by 31% (ZB2: 14.8 ± 1.2 U/mL vs. Control: 11.3 ± 0.9 U/mL; *p* < 0.01; Figure 8A), and GSH-Px activity by 24% (ZB2: 132.6 ± 9.3 U/mL vs. Control: 107.1 ± 8.2 U/mL; *p* < 0.05; Figure 8B). Correspondingly, serum MDA concentration, a lipid peroxidation end-product reflective of oxidative stress, was significantly reduced in ZB2 mice (ZB2: 3.12 ± 0.28 nmol/mL vs. Control: 4.67 ± 0.39 nmol/mL; *p* < 0.01; Figure 8C), confirming that *L. lactis* ZB2 supplementation confers meaningful protection against systemic oxidative damage. At the intestinal tissue level, colonic SOD activity was significantly elevated in ZB2 mice compared with Controls (ZB2: 43.2 ± 3.4 U/mg protein vs. Control: 31.7 ± 2.9 U/mg protein; *p* < 0.05), while colonic MDA content was significantly reduced (ZB2: 1.84 ± 0.18 nmol/mg protein vs. Control: 2.73 ± 0.22 nmol/mg protein; *p* < 0.01; Figures 8E,F).

## 4 Discussion

The present study systematically evaluated the probiotic potential of lactic acid bacteria isolated from Zhejiang fermented bamboo shoots and characterized the *in vivo* health-promoting effects of a superior strain, *L. lactis* ZB2, in a healthy murine model. Our results demonstrate that this strain possesses robust gastrointestinal survival capacity, superior colonization-related properties, and broad antimicrobial activity *in vitro*, and can substantially reinforce intestinal barrier integrity, modulate immune homeostasis, reshape the gut microbiota toward a beneficial composition, elevate fecal SCFA output, and enhance systemic antioxidant defenses *in vivo*. Collectively, these findings provide initial evidence supporting the probiotic application of *L. lactis* strains from Zhejiang fermented bamboo shoots as functional food ingredients or health-promoting agents.

The high acid (>82% at pH 2.0) and bile salt tolerance (>76% at 1.0% ox gall) of *L. lactis* ZB2 are prerequisite traits for any viable probiotic candidate, ensuring sufficient viable cell delivery to the intestinal target site. The observed tolerance levels are comparable to or exceed those of widely studied strains such as LGG and previously reported *L. lactis* isolates from fermented foods (Stage et al., 2020; De Chiara et al., 2024). This may reflect adaptive evolutionary pressures exerted by the acidic, low-pH fermentation environment of bamboo shoots, which could have selected for strains with enhanced proton pump activity and bile salt hydrolase expression (Bustos et al., 2025). Cell surface hydrophobicity is considered one of the most reliable physicochemical proxies for mucosal adhesion, and the hydrophobicity value of 68.4% obtained for ZB2 indicates a favorable affinity for epithelial surfaces. Similarly, the robust auto-aggregation (64.2% at 24 h) and co-aggregation activity of ZB2 against all three indicator pathogens are consistent with competitive exclusion of enteric pathogens and colonization stability (De Chiara et al., 2024; Lee et al., 2024). The particularly high co-aggregation against *Salmonella Typhimurium* (44.7%) is of potential public health relevance given the prevalence of this pathogen in foodborne illness.

The central finding of the animal study is the significant reinforcement of intestinal barrier function by *L. lactis* ZB2. The reduced serum FITC-dextran flux, along with decreased circulating DAO, D-lactic acid, and LPS levels, collectively provide converging *in vivo* evidence for a tighter epithelial barrier in treated mice. DAO and D-lactic acid are widely utilized biomarkers of intestinal mucosal integrity, and their reduction in the ZB2 group indicates attenuated epithelial damage and improved barrier sealing function (Wang et al., 2025). The significant downregulation of serum LPS in ZB2 mice is particularly noteworthy, as elevated LPS is associated with a state of metabolic endotoxemia that can drive low-grade systemic inflammation (Violi et al., 2023). The molecular underpinnings of this barrier reinforcement were confirmed by the significant upregulation of *ZO-1*, Occludin, and Claudin-1 at the mRNA level, as these tight junction proteins constitute the primary molecular paracellular barrier between adjacent enterocytes (Kuo et al., 2022; Horowitz et al., 2023). Additionally, the marked upregulation of *Muc2*, the primary intestinal mucin, indicates that *L. lactis* ZB2 also promotes the mucus layer, which acts as an additional physical and chemical barrier against luminal threats (Birchenough et al., 2023; Gallego et al., 2023). These findings are mechanistically consistent with the *AMPK/MLCK* signaling pathway known to be activated by certain *L. lactis* strains (Liu et al., 2025), though the specific molecular pathways engaged by ZB2 warrant further mechanistic investigation.

The immune-modulatory profile elicited by *L. lactis* ZB2 is characterized by a balanced anti-inflammatory tilt rather than systemic immunosuppression, which is a hallmark of health-promoting probiotics. The elevation of serum IL-10 and TGF-β, both critical immunoregulatory cytokines associated with regulatory T cell (Treg) function, combined with reduced IFN-γ, indicates a shift from a Th1-dominant toward a more immunoregulatory Th1/Th2 balance (Chen, 2023). In the intestinal mucosa, the downregulation of *TNF-α*, *IL-6*, and *IL-1β*, alongside upregulation of *IL-10*, is indicative of a dampened mucosal inflammatory tone that can prevent low-grade inflammation from progressing to pathological states (Papoutsopoulou et al., 2021). The simultaneous upregulation of *Reg3γ* and *β-defensin 1* is mechanistically coherent with the elevated sIgA levels observed, as these antimicrobial peptides and sIgA together constitute the principal components of mucosal innate and adaptive immunity, respectively (Chakraborty et al., 2024; Gleeson et al., 2024; Palrasu et al., 2025). These data suggest that *L. lactis* ZB2 engages multiple immunological nodes to establish a fortified yet balanced mucosal immune landscape.

The microbiota-reshaping capacity of *L. lactis* ZB2 may represent an important indirect mechanism underlying many of the observed functional benefits. Although alpha diversity, as assessed by the Shannon index, did not differ significantly between groups (*p* > 0.05), the numerical trend toward greater diversity in ZB2-supplemented animals suggests a subtle enrichment of community evenness that warrants further investigation in larger cohorts. The OPLS-DA score plot indicated partial separation between CON and ZB2 groups; however, the model showed limited explanatory and predictive power (Q2Y = 0.101), and permutation testing confirmed the absence of significant predictive validity (pQ2 = 0.40). These results should therefore be interpreted with caution and likely reflect the small sample size rather than a robust compositional difference.

At the genus level, STAMP-based differential abundance analysis identified nine genera that were significantly enriched in the ZB2 group, providing mechanistic insight into the functional consequences of ZB2 administration. Among the most markedly altered taxa, *Lacrimispora* and *Anaerotignum* are obligate anaerobes capable of fermenting dietary fibers to produce butyrate and other short-chain fatty acids (SCFAs), which serve as primary energy substrates for colonocytes and exert potent anti-inflammatory effects through inhibition of histone deacetylases and activation of G-protein-coupled receptors (Parada Venegas et al., 2019; van der Hee and Wells, 2021). The concomitant enrichment of *Lawsonibacter* and *Neglecta*, both members of the order Lachnospirales, further reinforces the SCFA-producing potential of the ZB2-restructured microbiota, given the well-established role of this clade in saccharolytic fermentation and intestinal homeostasis (Vacca et al., 2020). Similarly, *Enterocloster* and *Faecalimonas*, which also belong to the broader Lachnospiraceae lineage, have been associated with mucosal immune modulation and the induction of local regulatory T-cell populations, suggesting that ZB2 may promote a mucosal microenvironment conducive to epithelial barrier reinforcement and immune tolerance (Beresford-Jones et al., 2025).

The significant elevation of *Acutalibacter* and *Schaedlerella* in the ZB2 group is also noteworthy. *Acutalibacter*, a relatively recently characterized genus originally classified within *Ruminococcaceae* and now placed in the family Acutalibacteraceae, has been identified as a dominant member of the healthy murine intestinal microbiota and its enrichment here may reflect a broader restoration of a commensal-associated microbial configuration (Lagkouvardos et al., 2016). *Schaedlerella*, originally identified in murine models as a commensal associated with colonization resistance, may contribute to competitive exclusion of opportunistic taxa through niche occupation and antimicrobial metabolite production (Soh et al., 2019). Furthermore, the enrichment of *Mediterraneibacter*, a genus encompassing former *Ruminococcus* species characterized by a well-documented capacity for mucin glycan degradation and fermentative production of short-chain fatty acids including acetate and formate, lends additional metabolic breadth to the fermentative capacity of the ZB2-restructured microbiota (Togo et al., 2018; Crost et al., 2023). It should be noted that several of these genera remain incompletely characterized, and their functional roles inferred here are largely extrapolated from phylogenetic relationships and limited culture-based studies; direct functional evidence in murine or human models is lacking for most. Taken together, the enrichment of these nine genera—predominantly belonging to the butyrate-producing Lachnospiraceae and related families—points to a coherent functional shift in the gut ecosystem following ZB2 supplementation, characterized by enhanced fermentative capacity, improved metabolic output, and a community structure associated with reduced inflammatory tone.

The substantial and selective elevation of butyrate (70% increase) and propionate (53% increase) in ZB2 mice, with no significant change in branched-chain fatty acids, suggests that the microbiota restructuring drove enhanced fermentation of dietary fiber rather than proteolytic fermentation (LaBouyer et al., 2022). Butyrate, the preferred energy source for colonocytes and a potent inducer of tight junction protein expression and mucin synthesis via histone deacetylase inhibition, is likely a key metabolic nexus connecting the microbiota-level changes observed here with the functional outcomes at the intestinal epithelium (Gasaly et al., 2021; Liang et al., 2022). Propionate, which plays important roles in gluconeogenesis and appetite regulation, was similarly elevated, adding metabolic relevance to the SCFA findings. This SCFA-centric mechanism is consistent with recently described microbiota–SCFA–tight junction axes reported for other *Lactococcus lactis* strains (Liu et al., 2025). The concurrent antioxidant and anti-inflammatory effects observed may reflect a shared regulatory mechanism, as oxidative stress and inflammation are known to mutually reinforce each other through NF-κB-mediated pathways (Bellanti et al., 2025); however, the directionality and causal relationships between these outcomes in the context of ZB2 supplementation remain to be determined.

## 5 Limitations and future directions

Several important limitations should be acknowledged. First, the study was conducted in healthy, unstressed mice, and the outcomes may differ under pathological conditions such as colitis, metabolic syndrome, or immunodeficiency—future studies utilizing disease models would more directly inform the therapeutic potential of ZB2. Second, the mechanistic pathways connecting ZB2 to the observed barrier, immune, and microbiota outcomes remain to be fully delineated; proteomic, transcriptomic, and metabolomic approaches would provide deeper mechanistic insight. Third, the use of 16S rRNA gene amplicon sequencing inherently precludes strain-level resolution of microbiota changes, as this marker gene typically resolves microbial communities only to the genus or species level. Furthermore, the functional capacities inferred from taxonomic shifts remain correlative; shotgun metagenomic sequencing combined with metatranscriptomic approaches would provide direct functional annotation and more granular mechanistic information. Fourth, given that most health effects were inferred from a single-dose, single-time-point design, dose–response and time-course studies are needed to establish optimal administration parameters. Fifth, the absence of a positive control group (e.g., a well-characterized probiotic strain such as *Lactobacillus rhamnosus* GG or a mucosal protective agent) limits direct comparative assessment of ZB2’s efficacy in reinforcing intestinal barrier function relative to established benchmarks. Future studies should incorporate appropriate positive controls to substantiate claims of superiority or equivalence.

Building upon the present findings, several research directions merit prioritization. First, mechanistic studies employing transcriptomic, proteomic, and metabolomic approaches should be undertaken to delineate the specific signaling pathways—including Nrf2-mediated antioxidant responses, AMPK/MLCK–tight junction axes, and TLR4/NF-κB inflammatory cascades—engaged by *L. lactis ZB2*. Second, ZB2 should be evaluated in relevant disease models (e.g., DSS-induced colitis, high-fat diet-induced metabolic syndrome) to directly establish its therapeutic utility. Third, whether ZB2’s antioxidant capacity derives from promotion of antioxidant-competent microbiota members, SCFA-mediated Nrf2 pathway activation, or direct production of antioxidant metabolites requires experimental resolution.

## 6 Conclusion

In conclusion, *L. lactis* ZB2, a novel strain isolated from Zhejiang fermented bamboo shoots, demonstrates comprehensive probiotic functionality both *in vitro* and in a healthy murine model. The strain reinforces intestinal epithelial barrier integrity through upregulation of tight junction proteins and mucin, calibrates immune homeostasis toward an anti-inflammatory profile, enriches beneficial gut microbiota members, boosts SCFA production—particularly butyrate—and enhances systemic antioxidant defenses. These pleiotropic benefits collectively establish *L. lactis* ZB2 as a promising functional food ingredient and preventive health agent. The present findings also underscore the value of Zhejiang fermented bamboo shoots as an underexplored reservoir of superior probiotic strains, providing a scientific foundation for their further biotechnological development and functional food application.

## Data Availability

The raw data for 16S rRNA sequencing is stored in the NCBI Sequence Read Archive (SRA) under registration number PRJNA1448186 (https://www.ncbi.nlm.nih.gov/bioproject/PRJNA1448186). Additional data related to this study can be obtained from the corresponding author upon request.
