# Supplementary material for: Lactococcus lactis ZB2 from Zhejiang fermented bamboo shoots enhance intestinal barrier and immune homeostasis in mice
Source: Front Microbiol. 2026 Jun 24;17:1847505. doi: 10.3389/fmicb.2026.1847505 (PMC13342161; doi:10.3389/fmicb.2026.1847505)
Supplement: Supplementary file 1 [file Supplementary_file_1.DOCX]

**Supplementary Table S1.** Primers used for gene expression analysis via real-time qPCR.

| Gene | Primer sequence (5′–3′) | Product length, bp |
| --- | --- | --- |
| *ZO-1* | F: AGGTCTTCGCAGCTCCAAGAGAAA | 187 |
|  | R: ATCTGGCTCCTCTCTTGCCAACTT |  |
| Claudin-1 | F: CCACCATTGGCATGAAGTGC | 181 |
|  | R: CTGGCATTGATGGGGGTCAA |  |
| Occludin | F: TTGAAAGTCCACCTCCTTACAGA | 129 |
|  | R: CCGGATAAAAAGAGTACGCTGG |  |
| Mucin2 | F: GTCTTCAGGAGCTCTGGTGG | 115 |
|  | R: TACCACTCCAGTCCACAGCA |  |
| *Tnf-α* | F: TCTTCTCATTCCTGCTTGTGG | 129 |
|  | R: ATGAGAGGGAGGCCATTTG |  |
| *Il-6* | F: CAAAGCCAGAGTCCTTCAGAG | 106 |
|  | R: AGCATTGGAAATTGGGGTAG |  |
| *Il-1β* | F: GCCCCAAAGAGATGAAGTGC | 237 |
|  | R: TTTCAAGGACGATGGGCTCT |  |
| *Reg3γ* | F: CTGCAAGACAGACAAGATGCT | 102 |
|  | R: GCAACTTCACCTTGCACCTG |  |
| *β-defensin 1* | F: CCAGCTGCCCATCTAATACC | 112 |
|  | R: AATCCATCGCTCGTCCTTTA |  |
| Beta-actin | F: CATTGCTGACAGGATGCAGAAGG | 138 |
|  | R: TGCTGGAAGGTGGACAGTGAGG |  |

*ZO-1* = tight junction protein 1; *Tnf-α* = tumor necrosis factor alpha; *Il-6* = interleukin 6; *Il-1β* = interleukin 1 beta.


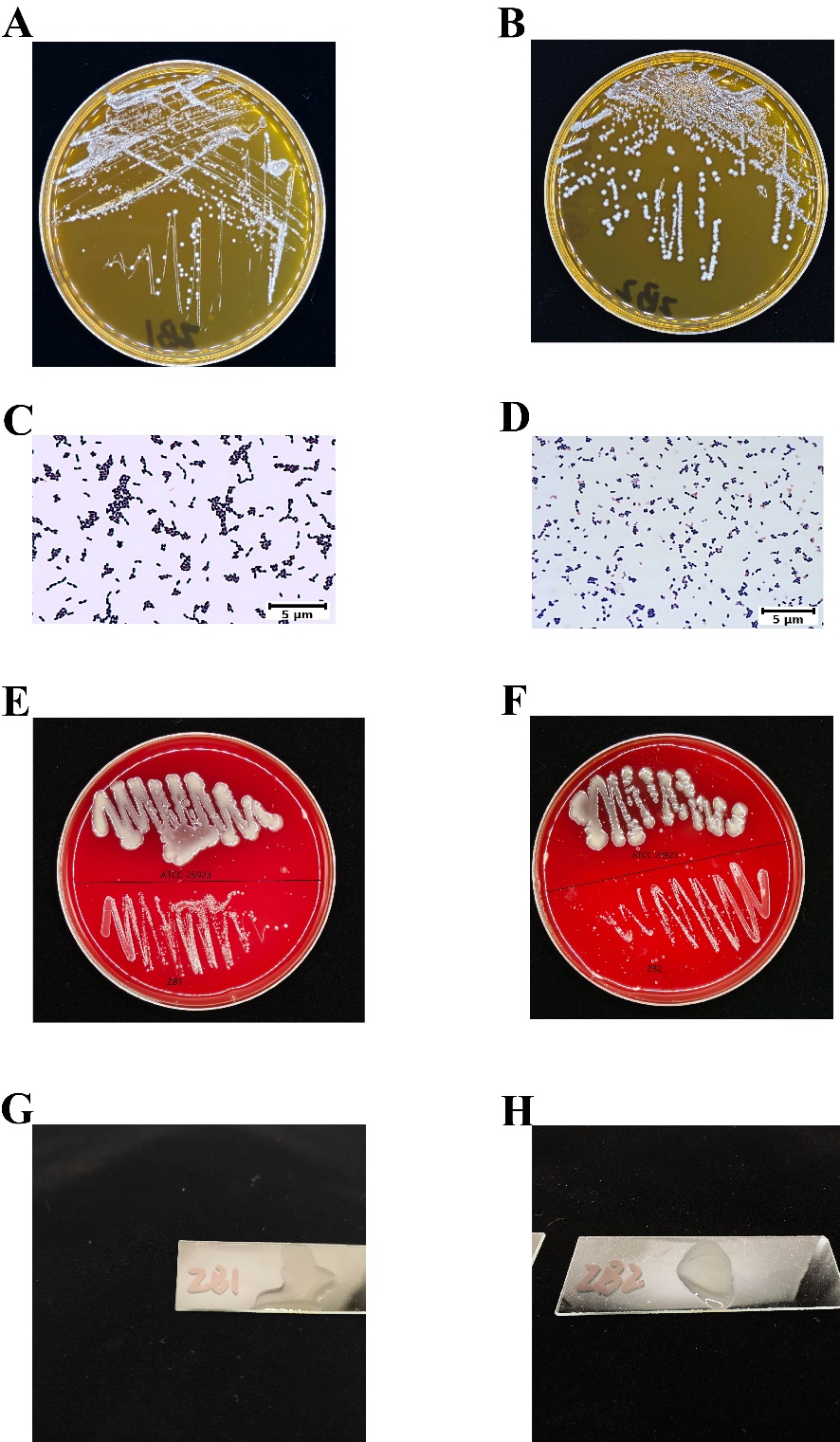


**Supplementary Figure S1.** **Morphological and biochemical characterization of** *Lactococcus lactis* **ZB1 and ZB2.** (A, B) Colony morphology of L. lactis ZB1 (A) and ZB2 (B) grown on M17 agar plates at 30°C for 48 h. Both strains produced circular, convex, smooth-edged, and cream-white colonies. (C, D) Gram staining micrographs of L. lactis ZB1 (C) and ZB2 (D). Cells appeared as Gram-positive (dark purple) cocci arranged predominantly in pairs or short chains. Scale bars = 5 μm. (E, F) Hemolytic activity of L. lactis ZB1 (E) and ZB2 (F) on sheep blood agar plates. Staphylococcus aureus ATCC 25923 was used as a positive control (upper half of each plate). Both ZB1 and ZB2 exhibited γ-hemolysis (no clearing zone around colonies), indicating the absence of hemolytic activity. (G, H) Catalase test of L. lactis ZB1 (G) and ZB2 (H). No bubble formation was observed upon the addition of 3% H₂O₂ to colonies on glass slides, confirming that both strains are catalase-negative, consistent with the taxonomic characteristics of the genus Lactococcus.
